# Phenotypic Tfh development promoted by CXCR5-controlled re-localization and IL-6 from radiation-resistant cells

**DOI:** 10.1007/s13238-015-0210-0

**Published:** 2015-09-24

**Authors:** Xin Chen, Weiwei Ma, Tingxin Zhang, Longyan Wu, Hai Qi

**Affiliations:** Tsinghua-Peking Center for Life Sciences, Tsinghua University, Beijing, 100084 China; Laboratory of Dynamic Immunobiology, Institute for Immunology, School of Medicine, Tsinghua University, Beijing, 100084 China

**Keywords:** Tfh, CXCR5, Bcl6, radiation-resistant cell, IL-6

## Abstract

How follicular T-helper (Tfh) cells develop is incompletely understood. We find that, upon antigen exposure *in vivo*, both naïve and antigen-experienced T cells sequentially upregulate CXCR5 and Bcl6 within the first 24 h, relocate to the T-B border, and give rise to phenotypic Bcl6^+^CXCR5^+^ Tfh cells before the first cell division. CXCR5 upregulation is more dependent on ICOS costimulation than that of Bcl6, and early Bcl6 induction requires T-cell expression of CXCR5 and, presumably, relocation toward the follicle. This early and rapid upregulation of CXCR5 and Bcl6 depends on IL-6 produced by radiation-resistant cells. These results suggest that a Bcl6^hi^CXCR5^hi^ phenotype does not automatically define a Tfh lineage but might reflect a state of antigen exposure and non-commitment to terminal effector fates and that niches in the T-B border and/or the follicle are important for optimal Bcl6 induction and maintenance.

**Electronic supplementary material:**

The online version of this article (doi:10.1007/s13238-015-0210-0) contains supplementary material, which is available to authorized users.

## INTRODUCTION

Tfh cells are functionally distinguished by their ability to deliver contact-dependent help to B cells inside the follicle (Crotty, 2011; Vinuesa and Cyster, 2011). Intrinsic T-cell expression of the transcriptional repressor Bcl6 is required for Tfh development and for germinal center (GC) formation (Johnston et al., 2009; Nurieva et al., 2009; Yu et al., 2009). How Bcl6 regulates Tfh development is not yet clear (Qi et al., 2014). One proposal is that Bcl6 drives CXCR5 upregulation, which subsequently promotes follicular localization of activated T cells. Consistent with this idea, Bcl6 overexpression in T cells *in vitro* downregulates multiple microRNA species such as the mir-17~92 cluster that potentially suppress CXCR5 expression, leading to increased CXCR5 transcript levels (Yu et al., 2009). However, genetic ablation of mir-17~92 impairs rather than promotes Tfh development and GC formation (Baumjohann et al., 2013; Kang et al., 2013). No evidence yet indicates direct Bcl6-mediated regulation of *Cxcr5* gene expression, and Bcl6-deficient T cells can upregulate CXCR5 under certain conditions (Liu et al., 2012), whereas the transcription factor Achaete-Scute homologue 2 (Ascl-2) directly binds to the *Cxcr5* locus and triggers CXCR5 upregulation (Liu et al., 2014). An alternative scenario is that Bcl6 drives a comprehensive Tfh developmental program of which CXCR5 upregulation is a manifestation (Crotty, 2014; Hatzi et al., 2015; Ueno et al., 2015). Consistent with this, T cells activated for 2 to 4 days *in vivo* are typically found to express Bcl6, likely under the influence of IL-6 (Nurieva et al., 2008; Harker et al., 2011; Choi et al., 2013), IL-12 (Ma et al., 2009; Schmitt et al., 2009; Nakayamada et al., 2011) and ICOS (Choi et al., 2011), while CXCR5^+^ T cells apparently only appear at later time points (Choi et al., 2011; Kerfoot et al., 2011; Kitano et al., 2011). Bcl6^+^CXCR5^+^ cells are located at the T-B border (Kerfoot et al., 2011; Kitano et al., 2011) and require DC- but not B cell-mediated antigen presentation (Deenick et al., 2010; Choi et al., 2011; Goenka et al., 2011; Kerfoot et al., 2011). In a kinetic study, dividing T cells were found to express high levels of Bcl6 2 days after immunization, while they did not express CXCR5 until approximately one day later (Baumjohann et al., 2011). Therefore, the Tfh developmental program is thought to follow a typical Th effector differentiation paradigm in that accessory signals delivered by DCs drives expression of fate-determining transcription factors, which then orchestrate epigenetically inheritable lineage commitment through successive cell cycles (Zhu et al., 2010; Crotty, 2011). However, here we have examined the earliest kinetics of CXCR5 and Bcl6 expression by T cells activated *in vivo* and surprisingly found that T cells with a Bcl6^hi^CXCR5^hi^ phenotype arise before the first cell cycle and that CXCR5 is required for optimal Bcl6 upregulation.

## RESULTS

### The rapid appearance of phenotypic Tfh cells before cell divisions *in vivo*

**Figure 1:**
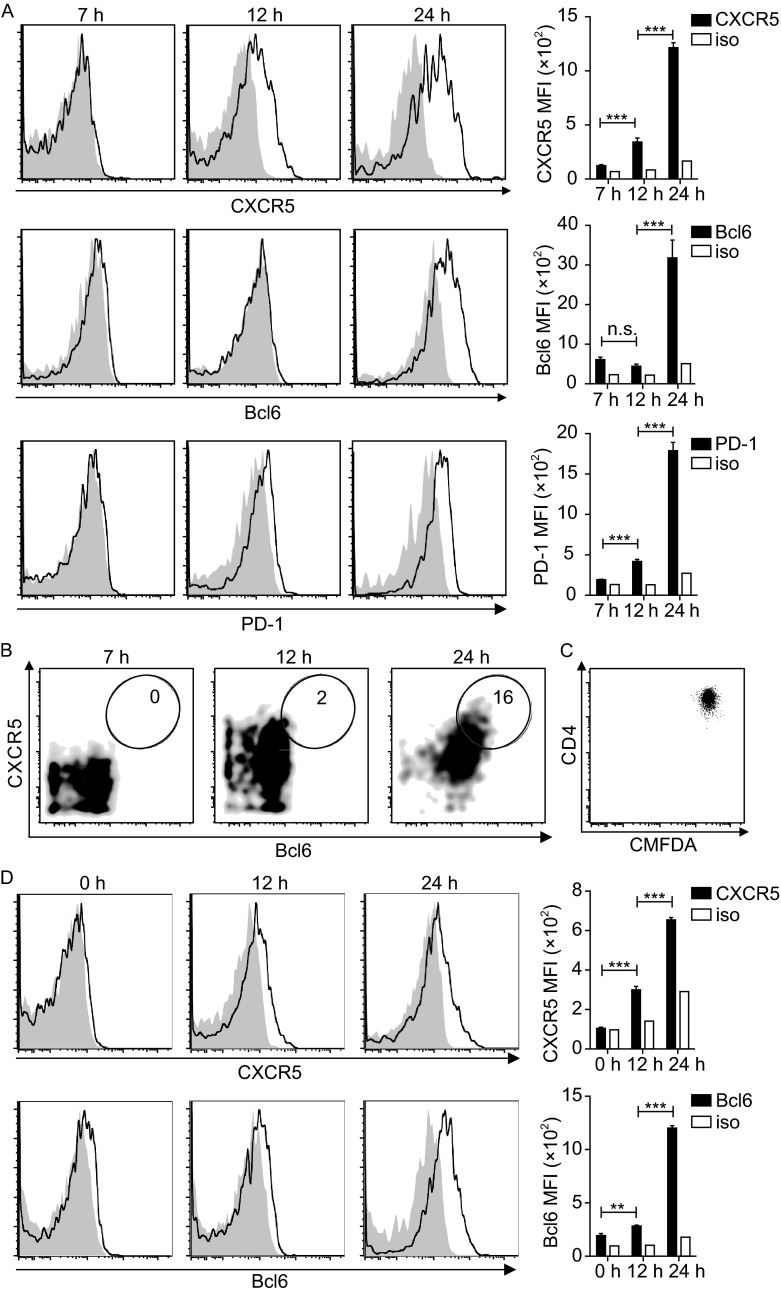
**BCL6** ^**hi**^ **CXCR5** ^**hi**^ **T cells emerge before the first division following activation** ***in vivo***. (A) Representative histograms and absolute MFI values of CXCR5, Bcl6, and PD-1 or corresponding isotype staining of CMFDA-labeled OT-II T cells at indicated times after OVA/alum/LPS immunization. (B) Representative bivariate displays of Bcl6 and CXCR5 staining patterns. (C) Representative CMFDA profile of OT-II T cells 24 h post immunization. (D) CXCR5, Bcl6, and PD-1 expression by CD25^-^CD62L^high^CD44^low^CD4^+^ OT-II T cells 24 h post immunization, measured as in (A). Data represent more than 3 independent experiments (3–4 mice per group). **P < 0.01; ***P < 0.001; n.s., not significant

To explore the earliest time point that T cells upregulate Bcl6, CXCR5 and acquire a Tfh phenotype following antigen activation *in vivo*, we examined OVA-specific OT-II CD4^+^ T cells in adoptive hosts at different time points after OVA immunization. As shown in Fig. 1A, by 12 h post immunization, OT-II T cells significantly upregulated CXCR5, while increase in Bcl6 expression was still minimal. PD-1 upregulation followed a similar kinetics as CXCR5. By 24 h, Bcl6 upregulation became evident, giving rise to an overt Bcl6^hi^CXCR5^hi^ population (Fig. 1A and 1B), even though virtually none of these cells divided (Fig. 1C). This rapid appearance of Tfh-like cells was not a recall response of memory cells potentially contaminating our T cell preparation, because CXCR5 and Bcl6 upregulation were also evident by 24 h when CD4^+^CD25^-^CD62L^high^CD44^low^ OT-II T cells were tested (Fig. 1D). It was not a peculiar behavior of naïve T cells, because OT-II T cells that were first activated *in vitro* also rapidly and sequentially upregulated CXCR5 and Bcl6 *in vivo* with an essentially identical kinetics after OVA immunization (Fig. S1). Together, these data suggest that CXCR5 and Bcl6 upregulation may be a stereotypic behavior of T cells responding to antigenic stimulation *in vivo* and that the appearance of a Bcl6^hi^CXCR5^hi^ Tfh phenotype *per se* could simply reflect acute antigenic activation rather than a differentiation program.

### Contribution of ICOS, CD40L and SAP signaling in early CXCR5 and Bcl6 upregulation

**Figure 2:**
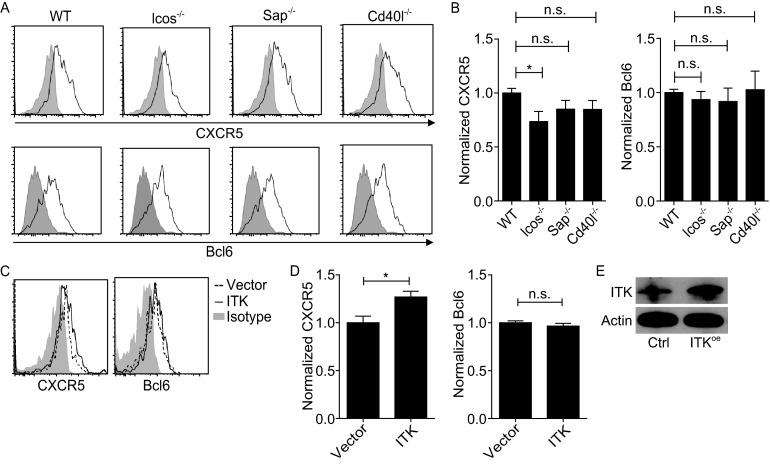
**ICOS and ITK signaling affects CXCR5 but not Bcl6 induction**. (A) Representative histograms of CXCR5 and Bcl6 expression by CMFDA-labeled OT-II T cells of indicated genotypes 24 h after OVA/alum/LPS immunization. Isotype staining was done with a mixture of WT, *Icos* ^-/-^, *Sap* ^-/-^, and *Cd40l* ^-/-^ cells. (B) Normalized CXCR5 and Bcl6 expression (the mean MFI of the WT group set as 1), shown as mean ± SEM of 10–13 recipient mice per group. Data are pooled from 4 independent experiments. (C and D) Representative histograms and normalized CXCR5 and Bcl6 expression (the mean MFI of the vector control group set as 1) on OT-II T cells transduced with the control or an ITK-overexpressing vector 24 h post immunization. (E) ITK levels in transduced cells as measured by Western blotting. Data are from two independent experiments involving 5 to 6 mice per group. *P < 0.05; n.s., not significant

It is well established that, by regulating T-B cell interactions and T cell-intrinsic signaling properties, ICOS, CD40L, and SAP are critical for Tfh development and GC formation (Crotty, 2011). To test whether ICOS, CD40L or SAP has a role in this rapid and early induction of the Tfh phenotype, we analyzed *Icos*^*-*/*-*^, *Cd40l*^*-*/*-*^ and *Sap*^*-*/*-*^ OT-II T cells in adoptive B6 hosts. As shown in Fig. 2A and 2B, normal CXCR5 upregulation required ICOS but not CD40L or SAP. Strikingly, early Bcl6 upregulation did not require any of these molecules. ICOS co-stimulation enhances calcium signaling and PI3K activation (Parry et al., 2003; Gigoux et al., 2009), with the Tec family kinase ITK serving as one intermediate (Berg et al., 2005; Nurieva et al., 2007). Consistent with the differential effect of ICOS on CXCR5 and Bcl6 upregulation, enforced ITK over-expression on T cells significantly enhanced early CXCR5 upregulation but had no effect on Bcl6 expression (Fig. 2C, 2D and 2E). Therefore, early CXCR5 and Bcl6 upregulation is a result of T cell receptor signaling and costimulation but not likely coupled in a simple regulatory circuit within T cells. CXCR5 upregulation is more sensitive to deprivation of ICOS co-stimulation than that of Bcl6.

### Early CXCR5 and Bcl6 expression correlate with T cell re-localization to the T-B border

**Figure 3:**
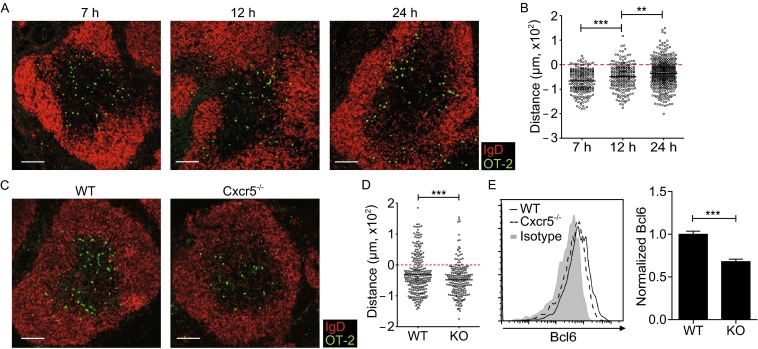
**CXCR5 is required for early T cell relocation to the T-B border and Bcl6 upregulation**. (A) Distribution patterns of OT-II T cells at indicated times after immunization. Scale bar, 100 μm. (B) Distances of individual cells to the T-B border, with negative and positive values indicating T-zone and follicular localization, respectively. Each symbol denotes one cell and lines denote mean values. A total of 223, 227, and 442 cells at 7, 12, 24 h respectively from at least 4 mice per time point were quantitated. (C) Distribution patterns of WT or *Cxcr5* ^-/-^ OT-II T cells 24 h post immunization. A total of 273 and 210 cells from WT and *Cxcr5* ^-/-^ group respectively and at least 2 mice per group were quantitated. Scale bar, 100 μm. (D) Distances of WT or *Cxcr5* ^-/-^ OT-II cells to the T-B border. (E) Representative histograms (left) and normalized Bcl6 expression (right) of WT or *Cxcr5* ^-/-^ OT-II T cells 24 h after OVA/alum/LPS immunization. Data are pooled from 3 independent experiments involving 11 mice per group. In (A) and (C), CD3 staining was omitted in the composite display for clarity. In (E), isotype staining was done with cells pooled from the respective two groups. **P < 0.01; ***P < 0.001

Although results presented above imply a stereotypic CXCR5 and Bcl6 upregulation following antigenic activation, it is important to note that mouse T cells activated *in vitro*, either by a combination of anti-CD3 and anti-CD28 stimulation or by antigen-pulsed dendritic cells or B cells, do not upregulate CXCR5 or Bcl6 with such kinetics (our unpublished data). These latter results suggest the importance of tissue microenvironment *in vivo* in permitting the rapid, antigen-triggered CXCR5 and Bcl6 upregulation. The fact that CXCR5 expression precedes overt Bcl6 induction raises the interesting question as to whether CXCR5-controlled T cell positioning in the tissue influences Bcl6 expression. As shown in Fig. 3A and 3B, within the first 7 h post immunization, OT-II T cells were uniformly distributed in the T cell zone. By 24 h, however, many OT-II T cells migrated to the T-B border and some were already inside the follicle. Such repositioning required CXCR5 upregulation, as *Cxcr5*^-/-^ OT-II T cells failed to do so (Fig. 3C and 3D). Interestingly, these *Cxcr5*^*-*/*-*^ OT-II T cells also failed to upregulate Bcl6 normally (Fig. 3E). Together, these data suggest environmental factors in the follicle or at the T-B border might positively promote Bcl6 expression.

### Early CXCR5 and Bcl6 expression are dependent on radiation-resistant cell derived IL-6

**Figure 4:**
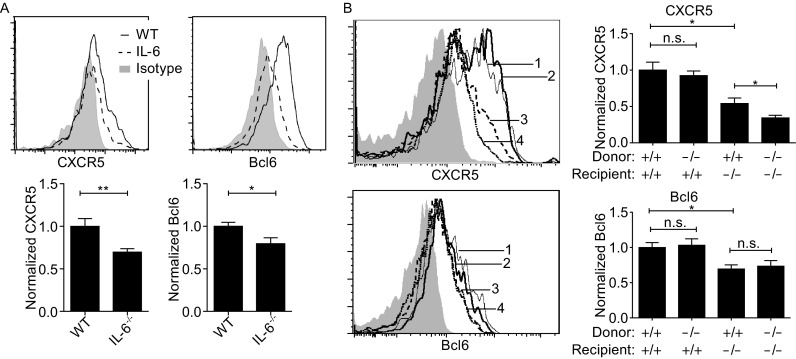
**IL-6 from radiation-resistant cells is critical for early CXCR5 and Bcl6 upregulation**. (A) Representative histograms (top) and normalized CXCR5 and Bcl6 expression (bottom) of OT-II cells one day after activation in WT or *Il-6* ^-/-^ recipients. Data are pooled from 2 independent experiments involving 5 or 6 mice per group. (B) Representative histograms (left) and normalized CXCR5 and Bcl6 expression (right) of OT-II cells one day after activation in chimeric hosts of indicated *Il-6* genotypes. 1: WT donor, WT recipient; 2: KO donor, WT recipient; 3: WT donor, KO recipient; 4: KO donor, KO recipient. Isotype control was show in gray. Cell mixture of the 4 groups was used for isotype control staining. Data are pooled from 2 independent experiments involving 5 to 9 mice per group. *P < 0.05; **P < 0.01; n.s., not significant

IL-6 promotes Tfh cell development and germinal center reaction *in vivo* (Nurieva et al., 2008; Wu et al., 2009), with DC-derived IL-6 believed to be primarily responsible (Harker et al., 2011; Vinuesa and Cyster, 2011; Choi et al., 2013). Consistent with the importance of IL-6, the rapid upregulation of CXCR5 and Bcl6 was impaired when OT-II cells were activated in *Il-6*^*-*/*-*^ recipients (Fig. 4A). We further constructed mixed bone-marrow (BM) chimera using wildtype or *Il-6*^-/-^ donors and recipients. Interestingly, OT-II cells normally upregulated CXCR5 and Bcl6 in wildtype recipients of *Il-6*^-/-^ donor BM cells under a condition of complete splenic DC replacement (Fig. S2), and reconstitution of *Il-6*^-/-^ recipients with wildtype BM cells led to somewhat improved but still significantly subdued upregulation of CXCR5 and Bcl6 on OT-II cells (Fig. 4B). These data suggest that IL-6 from radiation-resistant cells, possibly stromal cells, is essential for normal CXCR5 and Bcl6 induction on T cells, while BM-derived cells are not necessary or sufficient source of IL-6.

## DISCUSSION

This study reveals extremely rapid CXCR5 and Bcl6 upregulation by undivided T cells in the first 24 h after antigen exposure *in vivo*. Also using adoptive transfer of OT-II cells, Baumjohann et al. found that, at day 3.5 post immunization, Bcl6 was only upregulated in divided but not undivided T cells (Baumjohann et al., 2011). This apparent contradiction may be due to differences in anatomical locations examined or immunization routes utilized in the two studies. Interestingly, undivided OT-II cells detected at day 3.5 in our system also did not express Bcl6 (data not shown), suggesting above-mentioned factors cannot be solely responsible. Alternatively, individual T cells may be able to rapidly adjust CXCR5 and Bcl6 expression according to the strength of antigen stimulation and other environmental cues in the tissue, and lymphoid micro-environments in which antigen-activated T cells are located may change over time in content and distribution of such cues. Consistent with a role for environmental cues, CXCR5 is required for optimal Bcl6 upregulation in the very early phase, and CXCR5-mediated T-cell localization to the T-B border roughly coincides with the Bcl6 induction. We have further defined radiation-resistant cells as an important source of IL-6 that drives early CXCR5 and Bcl6 upregulation. Because stromal cells produce other cytokines and chemokines that can influence T cell functions (Malhotra et al., 2013), future studies of how stromal cells may regulate Tfh development are warranted. The fact that an apparent Tfh signature can be triggered in T cells even without cell division cautions the use of a CXCR5^hi^Bcl6^hi^ phenotype to automatically indicate a cell cycle-coupled, fate-commitment program as in models for Th1, Th2, and Th17 effector cell differentiation (Zhu et al., 2010). It remains possible that upregulation and maintenance of CXCR5 and Bcl6 represent a stereotypical behavior of T cells being exposed to antigen in a permissive tissue microenvironment and/or that a Tfh state can precede commitment to any effector differentiation. As cells of the Tfh phenotype are increasingly implicated in a large number of pathophysiological conditions including tumors and inflammatory diseases (Crotty 2014), the under-appreciated pattern of CXCR5 and Bcl6 expression on activated T cells as revealed in this study cautions us about assigning simple cause-effect relationship in situations involving potentially very complex interactions between T cells and their changing tissue environment *in vivo*.

## MATERIALS AND METHODS

### Mice and bone-marrow chimeras

C57BL/6 (Jax 664), μMT (Jax 2288), *Icos*^*-*/*-*^ (Jax 4859), GFP-expressing (Jax 4353), OVA_323–339_-specific T-cell receptor transgenic OT-II (Jax 4194), *Cd40l*^*-*/*-*^ (Jax 2770), *Il-6*^*-*/*-*^ (Jax 2650), *Cxcr5*^*-*/*-*^ (Jax 6659) mice were from the Jackson Laboratory. *Sap*^*-*/*-*^ mice were a kind gift of Dr. Pamela Schwartzberg (NIH). To construct chimeras, WT or *Il-6*^*-*/*-*^ mice were irradiated 2 × 500 rads with an X-ray source and then reconstituted with 4 × 10^6^ WT or *Il-6*^*-*/*-*^ bone marrow cells. Chimeras were used 8 weeks after reconstitution. All animal experiments have been conducted in accordance of governmental and institutional guidelines for animal welfare and approved by the Institutional Animal Care and Use Committee.

### Adoptive cell transfer and immunization

For naïve T cell transfer, each mouse received 1 × 10^6^–3 × 10^6^ OT-II CD4^+^ T cells isolated with the mouse CD4^+^ T cell isolation kit (Miltenyi Biotec) or further sorted as CD4^+^CD25^-^CD62L^high^CD44^low^ cells that were stained with 1 μmol/L CMFDA (Invitrogen). Recipient mice were immunized one day later. When previously activated T cells were used, 5 × 10^6^– 5 × 10^7^ OT-II T cells that were activated *in vitro* with plate-bound anti-CD3 and anti-CD28 for 4 days were injected to recipient mice 3 days prior to immunization. To immunize, 500 μg OVA protein (Sigma) in alum (Thermo Scientific) together with 5 μg LPS (Sigma) were intraperitoneally injected to recipient mice. For experiments involving ITK-over-expression, OT-II T cells were transduced with an MSCV-based vector that expresses GFP as a marker for cell identification by flow cytometry, as previously described (Xu et al., 2013). ITK overexpression was verified by Western blotting (anti-ITK antibody from Cell Signaling Technology).

### Flow cytometry

Single-cell suspensions were blocked with 20 μg/mL 2.4G2 (BioXcell) for 20 min and then stained with indicated primary antibodies for 90 min and secondary reagents for 30 min in PBS containing 1% FBS and 5 mmol/L EDTA. Staining reagents included PE-CF594 anti-CD4, PE-Cy7 anti-CD4, APC-Cy7 anti-CD19, and DyLight649 goat anti-hamster IgG antibody from Biolegend, purified anti-PD-1 from eBioscience, AlexaFluor 647 anti-Bcl6 (K112-91), biotinylated anti-CXCR5, PE- and BV421-labeled streptavidin from BD Biosciences. Isotype control antibodies included biotinylated Rat IgG2a AlexaFluor 647 Mouse IgG1 from BD Biosciences and purified hamster IgG from eBioscience. 7-AAD from Biotium was used to exclude dead cells. To detect intracellular Bcl6, cells were stained in the Foxp3/Transcription Factor Staining Buffer Set (eBioscience) after surface staining. To accurately measure CXCR5 and Bcl6 expression, care was taken to stain the same cell samples with the respective isotype control antibodies in parallel or, for certain experiments, to use an equal-proportion mixture of cells from different treatment groups for shared isotype control staining. Data were collected on BD LSR II or Aria III cytometers and analyzed with FlowJo software (TreeStar). When data from multiple independent experiments collected on different machines were pooled, we have normalized CXCR5 or Bcl6 MFI of individual samples against the mean MFI of the corresponding control group for each experiment.

### Immunohistochemistry

T-cell distribution patterns *in vivo* was analyzed on immunohistochemically stained splenic sections as previously described (Qi et al., 2006). Staining reagents included eFluor450 anti-CD3 (eBioscience) and AlexaFluor 647 anti-IgD (eBioscience). Sections were mounted with the ProlongGold Antifade reagent (Invitrogen) and imaged with an Olympus FV1000 microscope. To quantitatively measure positions of individual T cells in reference to the follicle, the T-B border was drawn as a line according to the CD3 staining patterns by a person blinded to sample identification, and the shortest distance from each T cell to the line was recorded, with negative and positive values indicating T-zone and follicular localization, respectively.

### Statistical analysis

For pairwise comparisons of endpoint means of experimental and control groups, two-tailed *t* tests were conducted using Prism (GraphPad).

## Electronic supplementary material

Supplementary material 1 (PDF 594 kb)
