# Supplementary material for: Phenotypic Tfh development promoted by CXCR5-controlled re-localization and IL-6 from radiation-resistant cells
Source: Protein Cell. 2015 Sep 24;6(11):825–32. doi: 10.1007/s13238-015-0210-0 (PMC4624673; doi:10.1007/s13238-015-0210-0)
Supplement: Supplementary file 1 — Supplementary material 1 (PDF 594 kb) [file 13238_2015_210_MOESM1_ESM.pdf]

Figure S1.

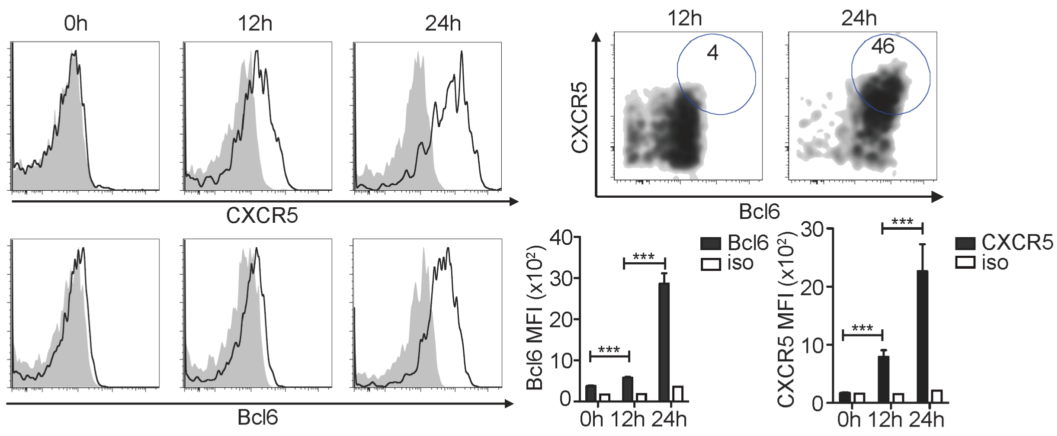

Figure S1. Levels of CXCR5, Bcl6, and PD-1 expression on previously activated OT-II T cells that were re-activated for 1 day in B6 recipients. The data are displayed as in Figs. 1A and 1B and represent 3 independent experiments (3-4 mice per group).

Figure S2.

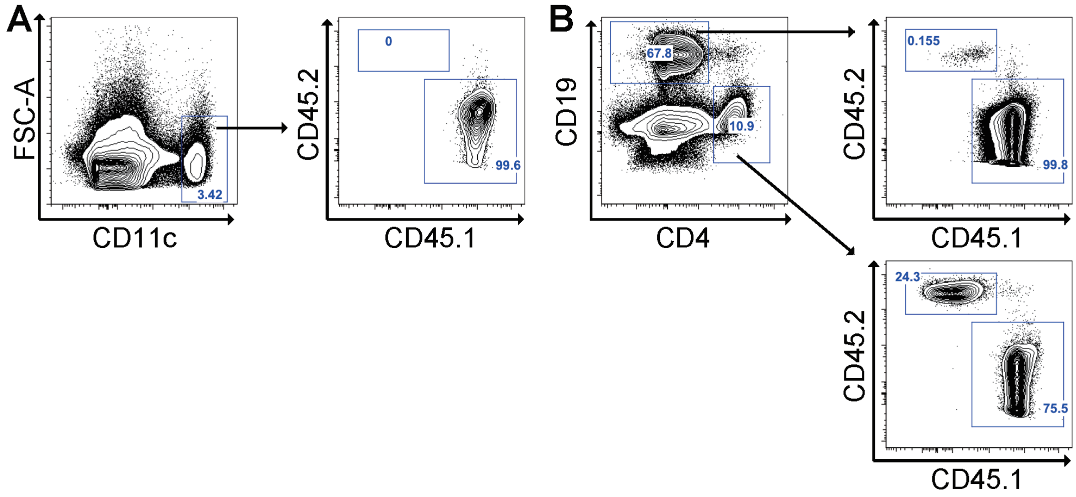

Figure S2. Replacement of DC and lymphocyte compartments by donor cells in radiation BM chimera. CD45.2 and CD45.1 contents in CD11c+ DC compartment (**A**), CD19+ B cell compartment and CD4+ T cell compartment (**B**) seven weeks after reconstitution of lethally irradiated CD45.2 B6 mice with CD45.1 bone marrows.
